# Safety and Digestibility of a Novel Ingredient, Brewed Lamb Protein, in Healthy Adult Dogs

**DOI:** 10.3390/ani15030427

**Published:** 2025-02-04

**Authors:** Stephen French, Chun-Yen Cochrane, Michael Faurot, Pernilla Audibert, Tomas Belloso, Dayakar V. Badri

**Affiliations:** 1 Pet Nutrition Center, Hill’s Pet Nutrition, Inc., Topeka, KS 66617, USA; stephen_french@hillspet.com (S.F.); chun-yen_cochrane@hillspet.com (C.-Y.C.); michael_faurot@hillspet.com (M.F.); 2 Bond Pet Foods, Inc., Boulder, CO 80301, USA; paudibert@bondpets.com

**Keywords:** brewed lamb protein, canine, digestibility, *Saccharomyces cerevisiae*, safety

## Abstract

Protein production through precision fermentation is an attractive option to reduce animal use in food production. This study focused on the safety and digestibility of food containing a test ingredient, brewed lamb protein (*Saccharomyces cerevisiae* expressing a lamb protein), at inclusion levels of 0% (control), 15%, 30%, or 40% were evaluated over 182 days in 40 dogs. No significant differences in body weight, body condition score, body composition, physical examination, food intake, or urinary parameters were observed among groups. Hematological and serum biochemical parameters were within reference ranges and were largely similar among groups. Levels of chloride, cholesterol, and homocysteine were significantly lower at some time points in dogs fed the test ingredient foods compared with the control food, but all remained within reference ranges. Whole blood taurine levels indicated no deficiency. Alanine aminotransferase levels were above the normal range at Day 177 in dogs in the control and 15% test ingredient groups. Fecal score was slightly decreased in dogs fed the 30% or 40% test ingredient foods, and fecal pH was decreased in dogs fed any of the test ingredient foods. Digestibility of most parameters was similar among foods, but apparent fat digestibility was significantly lower in foods with the test ingredient. Overall, these results indicate that the test ingredient is safe for use in dog food.

## Simple Summary

The environmental impact of food production, including food for pets such as dogs and cats, is an increasingly important issue. Animal protein production significantly contributes to this impact, highlighting the importance of developing more sustainable alternatives. In this study, the test ingredient, brewed lamb protein (*Saccharomyces cerevisiae* expressing a lamb protein), was used as the principal protein source in dog food. To examine the safety of this test ingredient, 40 dogs were divided into four groups, each fed one of the study foods (containing 0% [control food], 15%, 30%, or 40% of the test ingredient) over 26 weeks. Different measures of health (body weight, body condition score, body composition measures, physical examination parameters, food intake, blood biomarkers, and urinary parameters) indicated that there were few or no differences among groups. Levels of serum chloride, cholesterol, homocysteine, and whole blood taurine were lower in dogs fed the foods with the test ingredient compared to the dogs fed the control food, but all were within reference ranges. Digestibility was similar among all study foods, except that fat digestibility was lower in foods with the test ingredient. Altogether, these results indicate that the test ingredient is safe for use in dog food.

## 1. Introduction

Companion animal ownership is growing, with canine and feline populations in the United States of 89.7 million and 73.8 million, respectively [1]. Expanded populations means an increased demand for food, with corresponding effects on the environment. Despite the fact that pet food often incorporates animal byproducts, which are the materials from animal slaughter that are not consumed by humans, food for companion dogs and cats in the US still contributes to 25–30% of the environmental impact from animal production in terms of water, fossil fuels, land use, phosphate, and biocides [2]. Pet owners place high importance on environmental sustainability, and most are less likely to feed their pets a non-sustainable food when they are informed [3].

Most of the protein in dog and cat food is of animal origin [4] and contributes a significant amount to greenhouse gas emissions [5,6]. Among proteins of animal origin, beef (206 CO_2_ eq/g protein), lamb (172 CO_2_ eq/g protein), and veal (84 CO_2_ eq/g protein) have much higher average carbon footprints compared to chicken (25 CO_2_ eq/g protein) [7]. Reduced meat consumption is one way to increase the sustainability of food, considering that greenhouse gas emissions from the production of animal-based foods are twice as high as those from producing plant-based foods [8]. Vegetarian pet foods are increasingly popular among pet owners but often do not conform to the Association of American Feed Control Officials (AAFCO) or European Pet Food Industry Federation (FEDIAF) nutrient profiles [9,10]. This is often the result of inadequate supplementation of limiting amino acids from complementary protein sources. Thus, there is an opportunity for fermentation technology to produce alternative protein ingredients with controllable amino acid profiles and adequate digestibility [11].

Yeast in its conventional form has been extensively used in feed for poultry and livestock [12,13], and its use as a protein source in companion animal food has been increasing [14]. The US Food and Drug Administration has rated dried *Saccharomyces cerevisiae* and its extracts as generally recognized as safe for use in food [15]. In addition, *S. cerevisiae* is an AAFCO-approved ingredient [16], and the European Food Safety Authority determined that it is safe for use in dog food [17]. A few published short-term studies on dried yeast in dog foods indicate that it is not only safe for consumption but also has beneficial health effects. One study tested five foods with different primary protein sources, one of which was dried yeast (29% crude protein), over 14-day feeding periods [18]. Dogs were healthy throughout the study, fecal quality was ideal, serum chemistry profiles were within normal reference ranges, and the foods were highly digestible (>85%). Fecal butyrate, which confers many beneficial functions on health [19], was highest in the dogs fed the dried yeast food, and greater β-diversity was observed in the fecal microbiota in that group. Another study tested the inclusion of 10% dried yeast in dog food over a 10-day period and found no significant differences in nutrient digestibility or stool quality compared with a control food without yeast [20]. Food including brewer’s yeast with or without corn yeast showed similar total tract apparent digestibilities as a control food without yeast in dogs fed over 20-day periods [21]. In addition, supplementation with yeast conferred beneficial effects in intestinal functionality, as indicated by reduced fecal indole, increased fecal short-chain fatty acid concentrations, and greater abundance of beneficial fecal bacteria in dogs fed the yeast-supplemented food compared with those fed the control food.

Employing precision fermentation, a process in which microbes are employed to produce proteins and ingredients for food, is a burgeoning field, with yeast an attractive production host [11]. In fact, several proteins made through this process are currently in commercial production, including dairy and egg proteins [11]. Production of an animal protein via precision fermentation allows for incorporation of the animal protein into food products without the need for animal slaughter and without the environmental impact of raising animals for food production.

In the present study, food containing a novel test ingredient produced through precision fermentation, brewed lamb protein (*S. cerevisiae* expressing a lamb protein), as a protein source was tested in dogs. Retaining the yeast in the test ingredient delivers the documented nutritional benefits of yeast [12,13]. Lamb protein was chosen since it should meet AAFCO maintenance amino acid requirements [22], and lamb of animal origin has one of the highest carbon footprints of meats [7]. To evaluate the safety and nutritional adequacy of brewed lamb protein in dogs, this parallel-design study was undertaken over a 26-week period in 40 healthy dogs; independently, another study was conducted with a different cohort of 24 dogs for digestibility analyses.

## 2. Materials and Methods

### 2.1. Animals

For the safety study, a total of 40 clinically healthy adult dogs aged between 2.4 to 12.1 years and weighing 7.6 to 12.6 kg were selected from the Hill’s Pet Nutrition canine colony by an attending veterinarian. The number of subjects was chosen as described by the AAFCO-recommended protocol for safety studies. Dogs were clinically healthy on assessment, showing no clinically meaningful abnormalities on serum biochemical markers, complete blood cell count (CBC) blood tests, urinalyses, or physical examination, and had no known dietary allergies or appetite conditions. Dogs were also considered for their ability to cooperate with the procedures and sample collections required for this study. Dogs could not be pregnant or lactating, be less than one year old, have known disease conditions, or have a history of poor eating behavior.

Dogs were housed in rooms with windows for natural outdoor light, and automated LED lights maintained a cycle of 11 h light/13 h dark. The temperature in each room was maintained between 70 °F and 72 °F. Dogs were pair-housed in each room in a pen, with separate sleeping quarters within each pen. Water was available as needed. All dogs were allowed routine animal socialization with the other dogs, received enrichment activities with the animal care research technicians within the allocated room, and were allowed to play in the outdoor grassy area within the group. All dogs in the study were administered Tri-Heart Plus (Merck, Rahway, NJ, USA) monthly in accordance with the manufacturer’s recommendations for the prevention of heartworms.

This study protocol (CP1045b.0.0.0-A-C-D-ADH-MULTI-365-MULTI, approved on 15 September 2023 and valid until 5 July 2026) was approved by the Hill’s Institutional Animal Care and Use Committee (IACUC) followed by the Hill’s Animal Welfare Committee in accordance with the guide for the care and use of laboratory animals from the US National Research Council.

### 2.2. Test Ingredient Details

A comparative bioinformatic screen was used to identify highly abundant proteins in specific tissue types (e.g., muscle). Genes for these proteins were codon-optimized for expression in *S. cerevisiae* (strain CEN.PK113-7D) and chemically synthesized by Integrated DNA Technologies (Coralville, IA, USA). The synthesized sequence was placed between native *S. cerevisiae* regulatory elements (promoters and terminators), amplified by polymerase chain reaction, and introduced into a specific site in the *S. cerevisiae* genome. *S. cerevisiae* was then utilized to express a lamb protein under precision fed-batch fermentation conditions at about 30 °C and pH of approximately 5.0. The nutrient feed solution (primarily dextrose) and air were continuously added during fermentation. Fermentation was complete after all feeding solution had been added and when the concentration of biomass reached ideal conditions. The whole-cell biomass was then separated from the aqueous fermentation broth by centrifugation followed by heat treatment at a minimum of 80 °C for 30 min and spray dried to produce the dried, inactivated, whole-cell biomass from *S. cerevisiae* that contained a minimum of 50% protein, ≥10% of which was lamb protein (typically 10–15% total animal protein in the biomass by dry weight). The lamb protein was verified and quantified using an Orbitrap nano LC-MS (Thermo-Fisher Scientific, Waltham, MA, USA). The raw materials used to manufacture this finished ingredient are considered safe for use in companion animals.

The test ingredient, brewed lamb protein (Lot M-017 used in this study), typically contains mean ± SD 53.85 ± 1.4% protein, 27.66 ± 0.83% total dietary fiber (24.6 ± 0.46% insoluble fiber, 3.06 ± 0.59% soluble fiber), 5.1 ± 0.08% crude fat, 5.57 ± 0.45% moisture, and 5.69 ± 0.26% ash on an as-is basis. Protein provided from this ingredient is a complete protein because it meets the requirement of including all essential amino acids at the appropriate levels. The brewed lamb protein was manufactured in a food-grade facility, and both the product and the facility meet all regulatory and quality standards established by the U.S. Food and Drug Administration.

### 2.3. Food and Study Design

All foods met the AAFCO maintenance nutrient profile requirements and were formulated using the calculated modified Atwater mean ± SD isocaloric values of 3235.76 ± 87.14 kcal/kg [23]. The three test foods (15%, 30%, and 40% test ingredient) were formulated similarly to the control food except that the test ingredient substituted a portion of the egg protein (Table 1). Egg was chosen as the predominant protein source in the control food since it has high scores for digestible indispensable amino acids and protein-digestibility-corrected amino acids in dogs [24]. All four foods were made by extrusion processing at the Hill’s experimental food laboratory following Good Manufacturing Practice (GMP) guidelines. Hill’s experimental food laboratory follows typical GMP guidelines for quality control, including plant sanitation, food processing critical control points, lot tracking, and personal hygiene practices to ensure safe foodstuffs.

Nutrient analysis of the study foods was performed at Eurofins Nutrition Analysis Center (Des Moines, IA, USA) using standard Association of Official Agricultural Chemists (AOAC) methods [25].

**Figure 1:**
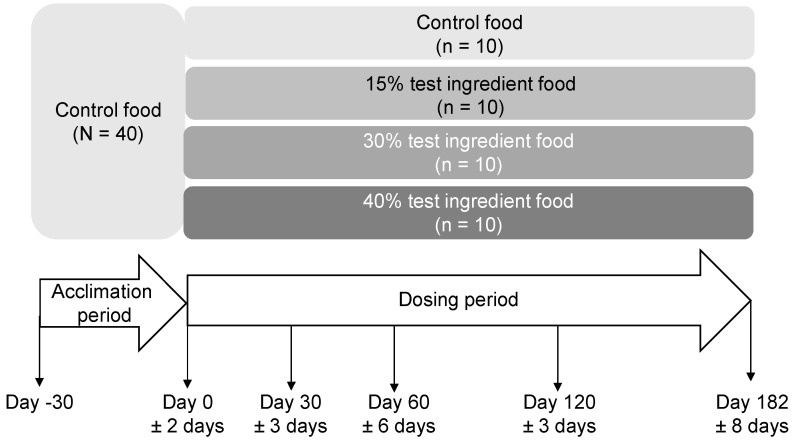
Safety study design and timeline in which dogs consumed foods containing 0%, 15%, 30% or 40% of the test ingredient (brewed lamb protein) for 182 days during the dosing period preceded by a 30-day acclimation period. Sample collections (blood, urine, and feces) and physical measurements were performed on Days 0, 30, 60, 120, and 182 of the dosing period. Body composition measurement was performed on Days 0, 60, and 182 of the dosing period.

The safety study was designed following AAFCO study protocols. All 40 dogs were fed the control food (0% inclusion of test ingredient) for 30 days in the acclimation phase (Figure 1). Dogs were then randomized to a group by random sampling without replacement from the four treatment choices (repeated 10 times for the 40 dogs); however, dog breed, age, sex, and body weight balance were considered in assigning the groups. Since the safety study followed a parallel design, treatment order was not a confounder. Each dog was fed their assigned food (control food or food with 15%, 30%*,* or 40% test ingredient) once daily each morning over a 30-min period. Twenty-six weeks was the dosing period as suggested by the AAFCO safety study protocol. Amounts of food were based on caloric energy requirements of the dogs, calculated based on their initial ideal body weight (BW) and body condition score (BCS). The group treatment allocation was known only by one of the corresponding authors (D.V.B.) and the test writer (C.S.) at all stages of the safety study.

### 2.4. Body Weight, Food Intakes, Body Condition Scores, Body Composition Analyses

BW was measured weekly over the 182-day safety study period and was used to calculate and adjust the food given to each dog to maintain an ideal BW throughout the study period. Food intakes were recorded daily by an automated feeding system. BCS was measured by an attending veterinarian on Days 0, 30, 60, 120, and 182 using a 5-point scale, with 3 being ideal and every integer point representing a 20% deviation in fat mass from the median (Table S1). Dogs were anesthetized during body composition measurements on Days 0, 60, and 182 using the Hologic QDR 4500 whole body scan machine (Hologic Inc., Marlborough, MA, USA). Total mass, lean mass, fat mass, bone mineral content, and bone mineral density were calculated for each dog.

### 2.5. Sample Collection and Analysis

In the safety study, fasted blood, feces (entire bowel movement), and first morning urine samples were collected on Days 0, 30, 60, 120, and 182. On those days, a physical examination that included heart rate as well as lymphatic (manual palpation of lymph nodes) and respiratory (stethoscope evaluation of lung sound and respiratory rate) evaluations were also performed for each dog by the attending veterinarian to assess heart, lung, and immune health. Blood samples were collected via jugular venipuncture in a vacutainer. Urine samples were collected via the cystocentesis method. Both blood and urine samples were collected by the attending veterinarian or trained animal care technician. CBC and white blood cell differential analyses were performed using the Sysmex XN-V 1000 Hematology Analyzer (Sysmex America, Inc., Lincolnshire, IL, USA). Serum biochemistry analysis was performed using a Cobas c(303) analyzer (Roche Diagnostics, Indianapolis, IN, USA).

Whole blood taurine levels were analyzed at Eurofins Nutrition Analysis Center (Des Moines, IA, USA) following a proprietary test method. Briefly, blood cells were lysed in three freeze/thaw cycles, and proteins were precipitated with the addition of sulfosalicylic acid. The sample was ultracentrifuged, and the supernatant was collected, filtered, and analyzed on a cation-exchange high-performance liquid chromatograph with post-column o-phthalaldehyde (OPA) derivatization. The resulting OPA-taurine molecule was detected using fluorescent emission.

Serum IgE levels were measured using the canine IgE ELISA kit (ab157700, Abcam, Cambridge, UK), and serum C-reactive protein (CRP) levels were measured with the dog CRP ELISA kit (E-40CRP, ICL Laboratories, Portland, OR, USA), both assessed by MLM Medical Minnesota Labs (Oakdale, MN, USA). Results were read on a FLUOstar Omega Multi-mode Microplate Reader (BMG Labtech, Ortenberg, Germany).

Urinalysis included measuring osmolality with the OsmoPRO Osmometer (Advanced Instruments, Norwood, MA, USA), pH by potentiometric method using a VWR B30PCI SympHony pH meter (VWR Inc., Radnor, PA, USA), and specific gravity using a Reichert temperature-controlled hand-held refractometer (Reichert Technologies, Depew, NY, USA). Bilirubin, ketones, and urobilinogen were measured with the IDEXX UA Strip (IDEXX laboratories, Westbrook, ME, USA). After sedimentation, casts were measured via microscopy with an Olympus CX41 (Olympus Life Sciences, Tokyo, Japan) under 40× magnification. About 0.5 mL of urine was processed to measure total protein and creatinine with a Cobas c(303) analytical unit.

Whole fecal samples were collected within 30 min of defecation after determining the fecal score on the Hill’s 1–5 scale (Grade 1 = watery diarrhea, no solid form, greater than 75% of stool is liquid; Grade 2 = texture, no defined shape, 50% liquid, 50% soft; Grade 3 = segmentation within fecal segments is rare, greater than 75% is soft, formed, not firm, less than 25% is liquid; Grade 4 = may see some segmentation, more linear and tube-like, greater than 50% of feces is firm and formed; Grade 5 = frequently segmented, greater than 85% of feces is firm and formed, cylindrical shape).

Whole fecal samples were homogenized with a Thinky Mixer (ARM-310, THINKY Laguna Hills, CA, USA) and frozen in cryovials at −80 °C until processing. Fecal calprotectin, IgA, and alpha-1-proteinase inhibitor (A1Pi) were also analyzed by MLM Medical Minnesota Labs. Fecal calprotectin levels were measured with the canine calprotectin ELISA kit (EQ13485DO-96, Lifeome, San Diego, CA, USA), IgA with the dog IgA ELISA kit (E44-104, Bethyl/Fortis Life, Montgomery, TX, USA), and A1Pi with the canine A1Pi SERPINA1 ELISA kit (MBS2801406, MyBiosource, San Diego, CA, USA); all were quantified with a FLUOstar Omega Multi-mode Microplate Reader (BMG Labtech).

### 2.6. Digestibility Analysis

Digestibility tests were performed using Hill’s dedicated digestibility panels. Four different digestibility panels of 6 dogs each (total of 24 dogs) were used to test the four study foods (0%, 15%, 30%, or 40% test ingredient). The digestibility study was performed with a 14-day acclimation period followed by a 5-day fecal collection period. Fecal sample collections and processing was performed using the AAFCO Dog and Cat Food Metabolizable Energy Protocols Method 1: Quantitative Collection [23] to assess the apparent digestibility of macronutrients. Analysis of fecal samples was performed at Eurofins Nutrition Analysis Center using standard AOAC methods.

Digestibility measures were calculated for each dog for each food tested and the average values are reported. Apparent digestibility measures were calculated for each dog by comparing the mass of each analyte based on the total weight of the food consumed and the mass of each analyte based on the total weight of the feces from the dog using the following formula:

Apparent digestibility (%) = ([intake − fecal output]/intake) × 100

True protein digestibility was measured by subtracting the endogenous metabolic fecal protein (fecal protein of non-dietary origin) estimate of 63 mg nitrogen/kg weight to the ¾ power [26] in the feces from the measured fecal protein concentration by using the following formula:

True protein digestibility (%) = (protein intake − [fecal protein − endogenous metabolic protein])/(protein intake) × 100

### 2.7. Statistical Analysis

Frequency tables by food and time were created for all categorial endpoints. Mean and standard deviation were calculated for all numerical endpoints. The numerical endpoints with measurements at multiple time points were further analyzed using both classic and robust linear mixed models using the lme4 [27] and robustlmm [28] packages. The mixed model included food, time, and their interaction (food × time) as fixed effects, the baseline measure at the end of the acclimation phase (Day 0) as a covariate, and dog as a random effect. The numerical endpoints without repeated measurements were analyzed using a linear model with food as a fixed effect. Post hoc analysis was also performed to determine differences between the test foods and control food at different time points using the emmeans package [29]. Since multiple comparisons were made for many endpoints, the *p*-values were adjusted by the Benjamini–Hochberg procedure to control the false discovery rate. An adjusted *p*-value ≤ 0.10 was considered a significant difference between foods for all primary endpoints except for digestibility and fecal score. For digestibility and fecal score, the cut-off adjusted *p*-value was 0.05. All statistical analyses were performed in R 4.4.1 [30].

## 3. Results

### 3.1. Nutrient Profiles of the Study Foods

All four study foods were similar in calories, ash, moisture, crude protein, and crude fat (Table 2). Crude fiber was highest in the control food and lowest in the food with 40% test ingredient. Total dietary fiber was similar across all four foods; however, insoluble and soluble fiber levels were different across study foods. Levels of both essential and non-essential amino acids were similar across all four foods and met the maintenance requirements of adult dogs as recommended by AAFCO [22].

### 3.2. Study Animals, Body Weight, Body Condition, Body Composition, and Food Intake

For the safety study, a total of 40 healthy adult dogs (20 neutered males, 20 spayed females; 37 beagles, 3 mixed breed), with a mean ± SD age of 6.70 ± 3.21 years (range, 2.08–12.08 years), an initial BW of 9.82 ± 1.42 kg, and initial BCS of 3.1 ± 0.38 participated in this study. Two dogs were withdrawn from the study due to medical conditions experienced during the study, and both subjects’ data were included in the data analyses until their removal from the feeding period. One dog was in the 15% test ingredient food group and was removed from the study on Day 133 due to active pancreatitis. Further diagnostic histopathology of the intestinal biopsy samples at Kansas State College of Veterinary Medicine determined that underlying undiagnosed chronic lymphocytic plasmacytic enteritis was the likely cause of the condition. The other dog withdrawn from the study was in the 40% test ingredient food group and was removed from the study on Day 78 due to low fecal scores. After switching to a veterinary food, the fecal scores of this dog improved to a 5 (normal). Two other subjects developed conditions during the study but continued in the study: one subject in the control group developed elevated liver enzyme levels and received Denamarin (Nutramax Laboratories, Lancaster, SC, USA) as a liver supplement, and another subject in the 30% test ingredient group developed allergic dermatitis and was prescribed lokivetmab (Cytopoint, Zoetis, Parsippany, NJ, USA) and oclacitinib (Apoquel, Zoetis) to treat allergic dermatitis. Both subjects showed improvement in their health after treatment, and no likely connection to the food has been determined. For the urinary endpoint analyses, collections for six subjects were missed on Day 120 and three were missed on Day 182 due to partial collection of urine volume by the cystocentesis method.

Physical examination outcomes, including heart rate, lymphatics, respiratory, and capillary refill time, were similar among all groups at all time points. Similarly, the lymphatic and respiratory conditions of all dogs were normal for all groups and time points examined in this study. No significant differences were observed in BW or BCS among groups (Table 3).

Body composition measures were assessed by dual energy X-ray absorptiometry (DEXA) to understand the food effect. No significant changes in total, fat, or lean mass or bone mineral content or density were observed among groups for the three time points measured in this study (Table S2). In addition, dogs consumed > 95% of the food offered for all study foods, and no significant differences were observed among food intakes (Table 3).

### 3.3. Hematological and Biochemical Parameters in Dogs That Consumed Foods with the Test Ingredient

All of the mean levels of hematological biomarkers were within reference ranges, and most were not significantly different among the groups except for nucleated red blood cell count (NRBC) and NRBC % on Days 30 and 58 (Table S3). However, all the levels remained within the normal reference range. The majority of samples had zero NRBC and NRBC % values; thus, even the presence of one to two non-zero observations implies statistically significant changes with no clinical significance.

The majority of the serum biochemical parameters did not differ among groups at any time points examined in this study (Table 4, Table S4). Mean levels of alanine aminotransferase (ALT), one of the markers of liver damage, were above the normal range at Day 177 in the control food and 15% test ingredient groups but not in the dogs that consumed the foods with higher levels of the test ingredient. Chloride levels were significantly lower in the dogs fed the foods with test ingredient compared to the control food on Days 30, 58, and 120 but not on Day 177, though all means were within the reference range. Cholesterol levels were significantly decreased in the dogs fed the 40% test ingredient food at all time points after Day 0 compared to the control group, and on Day 177, all dogs fed any of the foods with test ingredient had significantly lower cholesterol compared to the dogs fed control food. All mean cholesterol measurements at all time points were within the reference range. Finally, homocysteine levels were significantly lower for all time points except in the dogs fed the 30% test ingredient food at Day 177 and in dogs fed the 40% test ingredient food at Days 58, 120, and 177 compared to the control food, though all means were within the normal range.

CRP was only significantly different between the control and the 15% (increased) and 30% (decreased) test ingredient groups at Day 58 (Table S4). IgE did not differ among groups at any time point (Table 5, Table S4). All mean values of CRP and IgE were within the reference range and no dose response was observed. Whole blood taurine levels were significantly lower on Day 120 in the dogs fed any of the foods with test ingredient compared to the control food and also on Days 30 and 58 in dogs in the 40% test ingredient group (Table S4). However, no significant differences were observed among groups on Day 177.

### 3.4. Urinary Parameters in Dogs That Consumed Foods with the Test Ingredient

Physical urinary parameters and urinary analytes were within normal limits and did not significantly differ among groups at any time point examined in this study (Tables S5 and S6). Urinary crystal analyses were within the normal range for all time points except for one dog fed the 15% test ingredient food at Day 120 for calcium oxalate crystals (Table S7). No differences among groups were observed for urinary sedimentation analyses (Table S8). Urinary casts were absent for the majority of dogs in each group (Table S9).

### 3.5. Fecal Analysis in Dogs That Consumed Foods with the Test Ingredient

Fecal score was significantly decreased in the dogs that consumed the foods with 30% and 40% test ingredient compared to the dogs fed control food at all time points examined in this study (Table 6). However, no significant difference was observed in the dogs fed the 15% test ingredient food compared to those fed the control food. Fecal pH was significantly decreased in the dogs fed foods with any level of the test ingredient compared to the dogs fed control food at nearly all time points. Fecal calprotectin levels were only significantly higher in the dogs fed food with 40% test ingredient compared with the control food on Day 27, and fecal IgA levels were only significantly higher in the dogs fed food with 40% test ingredient compared with the control food Days 27 and 54. No significant differences were observed for A1Pi among the dogs in this study.

### 3.6. Digestibility of the Foods Containing the Test Ingredient

Dogs in the digestibility panels consisted of 21 beagles and 3 mixed breeds, 6 neutered males and 18 spayed females, with a mean ± SD age of 6.8 ± 2.1 years (range: 2.4–10.8 years). No significant differences were observed in true protein, apparent protein, crude fiber, or neutral detergent fiber digestibility measures among feces from dogs fed the study foods (Table 7). However, apparent fat digestibility was significantly decreased for the foods with 15%, 30%, or 40% test ingredient compared to the control food. Conversely, dry matter digestibility was significantly increased for the foods with 30% or 40% test ingredient compared to the control food. Metabolizable energy was similar among the four study foods.

## 4. Discussion

Two studies (26-week safety study and 19-day digestibility study) were undertaken to examine the safety and nutritional adequacy of a novel test ingredient, brewed lamb protein, in healthy adult dogs. Overall, there was no evidence of adverse events related to the product in dogs that consumed food including this test ingredient at various levels. The test ingredient at the evaluated inclusion levels did not affect BW, BCS (including lean mass), food intake, or other parameters of physical health. Hematology analyses were similar among groups at all time points except for NRBC, which was significantly higher in the control group compared with the other groups only on Day 30. Though lower, NRBC values for those fed the test foods remained in the reference range (0–0.03 k/µL). Serum biochemistry analytes, which included markers for liver health, renal health, protein levels, metabolism, and immune function, largely remained in a healthy range across all time points over the 182-day period for all study foods. Enzymatic activity of the liver marker ALT was above the reference range (22.3–90 U/L) at Day 177 in the control food and 15% test ingredient groups. These higher mean levels were due to one dog in each group that had elevated levels due to the administration of the medications carprofen and clomipramine, which can increase ALT enzymatic activity [32,33]. However, ALT levels were in the reference range for the dogs that consumed the foods with higher levels of the test ingredient, so the test ingredient did not cause the higher ALT levels in the control and 15% test ingredient groups. Despite similar chloride levels in all foods, serum chloride levels were significantly lower in dogs that consumed the test ingredient foods compared to the control food at several time points, but all were within the reference range (108.1–117 mmol/L), indicating a lack of clinical meaningfulness. One study suggested that short-chain fatty acids that are produced by gut microbes from dietary fiber in the colonic environment stimulate active chloride absorption in rats [34]. Thus, the impact of fiber in the test ingredient on short-chain fatty acid production and chloride absorption in the colonic environment warrants further investigation. Aside from the noted significant differences, there was no observed positive or negative trend based on the dosing of the test ingredient in the foods.

Cholesterol levels were significantly lower on Day 177 in the dogs fed foods with the test ingredient compared with the control food and also at earlier time points in dogs fed the 40% test ingredient food, though all levels were within the reference range (136–328 mg/dL). This observed decrease in cholesterol is likely due to the increased beta-glucans present in the yeast cell wall since decreases in serum cholesterol have previously been observed in various animals fed yeast beta-glucan, including obese dogs [35], mice [36], rats [37], and broiler turkeys [38], and in obese men [39]. One study of inclusion of 0.3% yeast hydrolysate in dog food for 14 days found significant decreases over time in serum cholesterol and triglycerides but increases in ALT and aspartate aminotransferase above the reference ranges in several dogs [40]. Of note, the use of German shepherds in that study versus mainly beagles (37/40 dogs) in the present study may account for the observed differences, as sometimes breeds can vary on a metabolic level.

Serum homocysteine levels were significantly lower at various time points in dogs fed the test ingredient foods compared to the control food. This result may indicate a beneficial effect of the test ingredient since high homocysteine levels are an indicator of deficiencies in B vitamins and folate [41,42] and are increased in dogs with mitral valve disease [43] and chronic kidney disease [44].

Whole blood taurine levels were lower at some time points from Days 0 to 177 across all test groups compared to the control, but no dose response was observed. At Day 120, whole blood from dogs fed any of the foods containing the test ingredient had significantly lower levels of taurine compared with blood from dogs fed the control food, and whole blood taurine levels were also lower on Days 30 and 58 in dogs that consumed the food with 40% test ingredient compared with the control food. Despite these significant differences from the control food, whole blood taurine levels remained well above the critical level of 150 nmol/mL, which is considered to be no known risk for taurine deficiency [31]. Dogs can synthesize taurine from methionine or cysteine [45], and the foods tested in this study show decreasing levels of methionine and cystine with increasing inclusion of the brewed lamb protein test ingredient, so this may explain the observation of the decreased whole blood taurine. Interestingly, a prior study found that dogs fed foods with lamb had the lowest whole blood taurine levels compared with those fed other protein sources [46]. In addition, there was no supplemented taurine in any of the foods tested in the present study. Given that whole blood taurine was lower in dogs fed the test ingredient foods compared with the control food, the lower homocysteine levels may indicate a general decrease in levels of sulfur amino acids and warrants further investigation.

Urinalysis, including physical parameters, chemistry, and microscopic sedimentation evaluation, of the dogs that consumed any of the study foods were in a healthy range across all time points, and no significant differences were observed among the treatments [47,48,49].

Fecal scores from dogs fed the food with the test ingredient at the inclusion levels of 30% and 40% were significantly lower compared with the dogs fed the control food at all time points. However, this result is not biologically significant because the mean fecal score for these foods was about 4 on the Hill’s 1–5 scale, meaning that >50% of feces were fully formed and were linear tube-like with some segmentation. The slight decrease in the fecal score is likely due to the yeast cell wall included in the test ingredient, which contributes soluble and insoluble fibers, leading to softer stools. The significant decrease in fecal pH in the dogs fed food that included the test ingredient at any level compared with the dogs fed the control food is also consistent with increased fiber from the yeast cell wall, as colonic fermentation of additional fiber lowers the fecal pH [50,51]. In other studies, slightly looser stools and/or decreased fecal pH have been observed in dogs fed dried yeast, yeast fermentation product, or yeast extract compared with control food [18,52,53]. No significant differences were observed among any study foods or time points in other fecal analytes, such as the gut immune markers calprotectin, IgA, and A1Pi, in the present study.

Digestibility is an important measure of food quality, and all digestibility parameters of the test foods in this study were acceptable. Apparent fat digestibility was significantly lower in dogs fed any of the foods with the test ingredient compared with the control food and decreased as the inclusion level of the test ingredient increased. This is consistent with decreased fat digestibility with increased dietary fiber in humans [54] and dogs [55]. Unlike these studies, however, the present study found no significant differences in true protein or apparent protein digestibility or in metabolizable energy among groups, whereas all were decreased with increasing fiber in the aforementioned studies [54,55].

There are a few limitations to this safety study. The current research was conducted using mostly beagles; thus, testing the effects of consumption in other breeds would add to the body of knowledge on this ingredient. Future research could further characterize the physiological effects of the addition of the test ingredient, such as examining short-chain fatty acids, fecal microbiota, and global serum and fecal metabolites. Data from this study may also inform future work on brewed lamb protein as a protein source in food for other animals.

## 5. Conclusions

The 26-week safety study found that foods with the test ingredient, brewed lamb protein, at the inclusion level of 15%, 30%, or 40% showed no adverse health measures in healthy dogs despite a few differences found in dogs consuming the food with the test ingredient compared to the control food. Specifically, BW, BCS, food intake, physical parameters, hematology, clinical chemistry, urinalyses, and fecal characteristics were largely unchanged compared with dogs fed the control food and/or were considered to be acceptable. Similarly, digestibility measures were largely unchanged except for the decrease in the apparent fat digestibility compared with dogs fed the control food. Thus, this test ingredient should be safe for use in canine nutrition.

## Figures and Tables

**Table 1:** Ingredients of foods used in the study.

		Food with Test Ingredient		
Ingredient, %	Control Food	15%	30%	40%
Brewed lamb protein (test ingredient)	0	15	30	40
Egg protein	24.63	15.48	6.22	0.10
Brewer’s rice	23.03	22.08	21.50	21.07
Whole wheat	23.03	22.08	21.50	21.07
Cellulose	11.21	7.08	2.89	0.10
Chicken fat	5.98	5.62	5.25	5.00
Dicalcium phosphate	3.03	2.00	2.00	2.00
Palatant	2.75	2.75	2.75	2.75
Lactic acid	1.20	1.20	1.20	1.20
Soybean oil	1.00	1.00	1.00	1.00
Sodium chloride	1.00	1.00	1.00	1.00
Potassium chloride	1.00	1.00	1.00	1.00
Natural flavor	0.75	0.75	0.75	0.75
Choline chloride	0.50	0.50	0.50	0.50
Calcium carbonate	0.44	2.00	2.00	2.00
Mineral premix	0.25	0.25	0.25	0.25
Vitamin premix	0.17	0.17	0.17	0.17
Antioxidant	0.04	0.04	0.04	0.04

**Table 2:** Nutrient profiles of the study foods on an as-fed basis.

		Food with Test Ingredient		
Analyte	Control Food	15%	30%	40%
Calories (modified Atwater), kcal/kg	3128.48	3192.07	3281.55	3340.95
Choline, ppm	3630.00	3720.00	3890.00	4060.00
Macronutrients				
Ash	6.01	6.81	7.06	7.29
Moisture	7.95	7.69	7.89	8.37
Crude protein	24.50	23.69	24.81	24.38
Crude fat	9.47	10.12	9.77	9.71
Crude fiber	7.80	4.80	2.50	0.90
NFE	44.27	46.89	47.97	49.35
Total dietary fiber	14.70	13.00	13.10	12.30
Insoluble fiber	14.20	11.70	11.30	10.10
Soluble fiber	0.50	1.30	1.80	2.20
Essential amino acids				
Arginine	1.31	1.34	1.40	1.35
Histidine	0.60	0.52	0.51	0.48
Isoleucine	1.25	1.23	1.29	1.26
Leucine	2.10	2.00	2.06	1.99
Lysine	1.70	1.71	1.85	1.81
Methionine	0.77	0.58	0.48	0.37
Phenylalanine	1.47	1.26	1.19	1.08
Threonine	1.09	1.10	1.18	1.15
Tryptophan	0.40	0.34	0.32	0.29
Valine	1.74	1.46	1.36	1.20
Non-essential amino acids				
Alanine	1.50	1.42	1.45	1.38
Aspartic acid	2.55	2.37	2.41	2.25
Cystine	0.70	0.49	0.36	0.27
Glutamic acid	3.70	3.50	3.53	3.39
Glycine	0.95	0.99	1.07	1.08
Proline	1.00	0.94	0.90	0.84
Serine	1.66	1.40	1.32	1.17
Taurine, ppm	<100	<100	<100	<100
Tyrosine	0.59	0.69	0.73	0.67
Fatty acids				
Omega-3 Sum	0.15	0.16	0.13	0.15
Omega-6 Sum	2.08	2.13	1.97	1.87
Omega-6: Omega-3	13.87	13.31	15.15	12.47
EPA (C20:5)	<0.02	<0.02	<0.02	<0.02
DHA (C22:6)	<0.02	<0.02	<0.02	<0.02
Minerals				
Calcium	1.00	1.18	1.27	1.09
Phosphorus	0.83	0.74	0.94	1.01
Sodium	0.61	0.52	0.51	0.48
Chloride	1.36	1.35	1.33	1.36
Potassium	0.82	0.93	1.13	1.16
Magnesium	0.09	0.10	0.12	0.12
Sulfur	0.48	0.37	0.31	0.27
Zinc, ppm	425.00	398.00	445.00	445.00

Presented units are percentages unless otherwise indicated. DHA, docosahexaenoic acid; EPA, eicosapentaenoic acid; NFE, nitrogen-free extract; ppm, parts per million.

**Table 3:** Body weight, body condition score, and food intakes of dogs at the end of the study.

		Food with Test Ingredient		
Parameter	Control Food	15%	30%	40%
Body weight, kg	9.89 ± 1.52	9.68 ± 1.25	9.73 ± 1.75	9.65 ± 1.26
Body condition score	3.1 ± 0.32	3.11 ± 0.33	3.0 ± 0	3.0 ± 0
Food intake, %	96.7 ± 5.7	95.7 ± 6.6	95.8 ± 6.6	97.4 ± 2.1

Values are mean ± standard deviation. Each group contained 10 dogs, except the 15% and 40% test ingredient groups contained 9 dogs.

**Table 4:** Selected serum chemistry parameters from dogs fed the study foods.

	Reference Range		Food with Test Ingredient		
Parameter		Control Food	15%	30%	40%
ALP, U/L	17.4–135.2				
Day 0		114.47 ± 111.50	120.17 ± 70.52	141.96 ± 101.49	128.84 ± 68.0
Day 177		98.98 ± 88.08	114.63 ± 119.92	120.31 ± 57.21	96.20 ± 51.36
ALT, U/L	22.3–90				
Day 0		78.13 ± 61.16	84.29 ± 80.33	69.88 ± 39.66	49.29 ± 20.46
Day 177		**110.87 ± 188.02**	**130.49 ± 256.12**	52.58 ± 30.39	34.14 ± 11.72
AST, U/L	22–46				
Day 0		45.62 ± 23.28	37.43 ± 7.76	**46.55 ± 19.42**	35.34 ± 7.90
Day 177		40.88 ± 24.85	44.92 ± 37.49	34.05 ± 9.95	29.69 ± 6.08
Albumin, g/dL	2.97–4.04				
Day 0		3.42 ± 0.21	3.45 ± 0.26	3.37 ± 0.35	3.54 ± 0.20
Day 177		3.29 ± 0.19	3.47 ± 0.16	3.35 ± 0.43	3.40 ± 0.20
Albumin:globulin ratio	1.2–2.46				
Day 0		1.80 ± 0.26	1.63 ± 0.29	1.68 ± 0.39	1.75 ± 0.36
Day 177		1.61 ± 0.21	1.54 ± 0.26	1.58 ± 0.32	1.54 ± 0.29
BUN, mg/dL	8–25.1				
Day 0		14.37 ± 1.73	13.86 ± 2.40	14.62 ± 2.92	14.01 ± 2.61
Day 177		14.53 ± 1.54	12.52 ± 1.54	15.50 ± 2.71	13.90 ± 2.20
Bilirubin total, mg/dL	0–0.11				
Day 0		0.07 ± 0.01	0.06 ± 0.02	0.08 ± 0.03	0.07 ± 0.02
Day 177		0.07 ± 0.01	0.07 ± 0.02	0.05 ± 0.02	0.06 ± 0.02
Calcium, mg/dL	9.2–10.7				
Day 0		9.76 ± 0.29	9.69 ± 0.30	9.70 ± 0.48	9.95 ± 0.31
Day 177		9.92 ± 0.20	10.02 ± 0.16	10.08 ± 0.45	10.23 ± 0.31
Chloride, mmol/L	108.1–117				
Day 0		114.58 ± 1.29	114.06 ± 1.11	114.02 ± 2.02	113.92 ± 2.02
Day 177		113.29 ± 1.12	111.41 ± 1.16	111.52 ± 1.47	111.53 ± 1.93
Cholesterol, mg/dL	136–328				
Day 0		217.4 ± 52.85	209.60 ± 72.84	193.7 ± 50.65	242.7 ± 58.35
Day 177		253.1 ± 63.41	206.0 ± 54.12 *	200.6 ± 63.52 *	220.44 ± 62.72 *
Creatinine, mg/dL	0.5–1.0				
Day 0		0.63 ± 0.10	0.58 ± 0.10	0.56 ± 0.08	0.63 ± 0.11
Day 177		0.71 ± 0.10	0.67 ± 0.12	0.67 ± 0.10	0.72 ± 0.10
Fructosamine, µmol/L	228–314				
Day 0		284.4 ± 24.68	303.10 ± 37.49	289.40 ± 20.84	298.10 ± 22.05
Day 177		278.0 ± 21.23	296.22 ± 42.76	277.7 ± 28.16	279.67 ± 25.49
GGT, U/L	2.18–7.4				
Day 0		2.58 ± 2.07	**0.01 ± 3.47**	**0.78 ± 2.68**	**2.17 ± 3.11**
Day 177		**0.67 ± 3.59**	**0.54 ± 7.80**	**0.43 ± 0.85**	**0.99 ± 2.04**
Globulin, g/dL	1.48–2.6				
Day 0		1.92 ± 0.20	2.16 ± 0.31	2.08 ± 0.34	2.08 ± 0.35
Day 177		2.06 ± 0.18	2.29 ± 0.31	2.16 ± 0.29	2.26 ± 0.31
Glucose, mg/dL	73.6–112.5				
Day 0		92.69 ± 9.11	93.86 ± 8.39	94.98 ± 6.64	91.53 ± 8.47
Day 177		88.59 ± 5.01	90.79 ± 5.02	98.16 ± 7.43	89.43 ± 6.92
Homocysteine, µmol/L	5.0–26.38				
Day 0		14.04 ± 7.89	16.41 ± 11.62	18.41 ± 9.19	12.50 ± 5.09
Day 177		11.52 ± 4.59	8.05 ± 1.17 *	9.73 ± 1.69	9.83 ± 2.61
Phosphorus, mg/dL	2.2–4.5				
Day 0		3.45 ± 0.39	3.62 ± 0.33	3.65 ± 0.51	3.56 ± 0.40
Day 177		3.29 ± 0.37	3.40 ± 0.33	3.53 ± 0.39	3.51 ± 0.48
Triglycerides, mg/dL	25.0–133.0				
Day 0		68.33 ± 11.09	96.27 ± 34.36	80.11 ± 24.81	78.03 ± 52.39
Day 177		71.64 ± 20.65	81.37 ± 24.39	76.29 ± 24.76	90.79 ± 32.62

Data are presented as mean ± standard deviation. Boldface indicates values outside the reference range. Each group and time point consisted of 10 dogs, except for 9 dogs in the groups fed the 15% or 40% test ingredient foods at Day 177. ALP, alkaline phosphatase; ALT, alanine aminotransferase; AST, aspartate aminotransferase; BUN, blood urea nitrogen; GGT, gamma-glutamyl transferase. * Adjusted *p* ≤ 0.1 vs. control food.

**Table 5:** C-reactive protein, IgE, and whole blood taurine at Days 0 and 177 from dogs fed the study foods.

	Reference Range		Food with Test Ingredient		
Parameter		Control Food	15%	30%	40%
CRP, µg/mL	<10				
Day 0		6.22 ± 4.85	4.68 ± 2.79	3.87 ± 2.01	4.57 ± 3.59
Day 177		7.44 ± 9.0	4.88 ± 3.87	4.62 ± 7.07	3.40 ± 3.16
IgE, µg/mL	1–41				
Day 0		11.91 ± 12.86	19.56 ± 19.76	22.02 ± 18.07	10.71 ± 10.46
Day 177		14.01 ± 11.97	18.85 ± 14.99	22.12 ± 18.18	13.14 ± 11.07
Whole blood taurine, nmol/mL	>200 *				
Day 0		306.3 ± 29.95	296.70 ± 45.57	285.30 ± 42.93	287.70 ± 56.35
Day 177		219.0 ± 24.34	**190.89 ± 27.65**	**180.80 ± 16.98**	**177.56 ± 24.72**

Data are presented as mean ± standard deviation. Boldface indicates values outside the reference range. Each group and time point consisted of 10 dogs, except for 9 dogs in the groups fed the 15% or 40% test ingredient foods at Day 177. CRP, C-reactive protein; Ig, immunoglobulin. * <150 nmol/mL is considered to be taurine-deficient [31].

**Table 6:** Fecal analytes from dogs fed the study foods.

		Food with Test Ingredient		
Parameter	Control Food	15%	30%	40%
Fecal score, 1–5 scale				
Day 0	4.90 ± 0.32	4.80 ± 0.63	5.0 ± 0	5.0 ± 0
Day 27	4.65 ± 0.47	4.05 ± 0.96	3.50 ± 1.0 *	3.40 ± 1.07 *
Day 54	4.80 ± 0.42	4.35 ± 0.88	3.94 ± 0.81 *	3.65 ± 0.94 *
Day 117	4.60 ± 0.52	4.60 ± 0.52	3.35 ± 0.53 *	3.11 ± 0.60 *
Day 174	4.80 ± 0.48	4.67 ± 0.50	3.70 ± 0.98 *	3.83 ± 0.61 *
pH				
Day 0	6.13 ± 0.14	6.20 ± 0.20	6.20 ± 0.23	6.18 ± 0.25
Day 27	6.04 ± 0.13	5.78 ± 0.25 *	5.52 ± 0.20 *	5.52 ± 0.19 *
Day 54	6.03 ± 0.18	5.77 ± 0.17 *	5.61 ± 0.25 *	5.47 ± 0.18 *
Day 117	6.05 ± 0.19	5.65 ± 0.18 *	5.65 ± 0.25 *	5.40 ± 0.23 *
Day 174	5.84 ± 0.34	5.59 ± 0.20 *	5.61 ± 0.20	5.50 ± 0.17 *
Calprotectin, ng/g				
Day 0	45.40 ± 35.89	67.78 ± 51.53	95.26 ± 65.0	72.40 ± 64.96
Day 27	63.72 ± 50.32	148.93 ± 294.83	79.57 ± 68.85	298.34 ± 370.44 *
Day 54	22.56 ± 27.46	74.10 ± 77.48	106.98 ± 110.66	84.97 ± 71.99
Day 117	62.52 ± 54.48	87.82 ± 86.49	122.06 ± 269.97	49.37 ± 55.99
Day 174	79.31 ± 80.43	103.13 ± 80.66	86.65 ± 88.66	107.50 ± 97.54
IgA, mg/g				
Day 0	7.19 ± 4.99	5.83 ± 4.96	4.78 ± 3.40	4.47 ± 3.07
Day 27	4.35 ± 3.02	6.24 ± 5.10	6.99 ± 6.84	9.05 ± 5.13 *
Day 54	4.72 ± 3.45	4.53 ± 3.67	8.28 ± 8.25	9.42 ± 5.76 *
Day 117	9.82 ± 10.10	4.57 ± 1.82	13.84 ± 11.55 *	10.27 ± 9.27
Day 174	6.39 ± 5.04	6.34 ± 6.94	12.0 ± 15.09	7.19 ± 5.06
Alpha 1-proteinase inhibitor, µg/g				
Day 0	29.98 ± 18.42	57.74 ± 56.93	66.07 ± 59.82	57.11 ± 71.94
Day 27	59.54 ± 63.59	146.92 ± 338.50	65.63 ± 86.92	288.09 ± 507.97
Day 54	33.40 ± 47.97	93.07 ± 99.11	108.02 ± 130.68	48.84 ± 50.30
Day 117	57.76 ± 41.76	78.67 ± 96.86	147.25 ± 365.98	47.15 ± 49.23
Day 174	68.73 ± 65.86	74.65 ± 67.73	84.11 ± 72.39	80.74 ± 63.43

Data are presented as mean ± standard deviation. Each group and time point consisted of 10 dogs, except for 9 dogs in the groups fed the 15% or 40% test ingredient foods at Day 174. Ig, immunoglobulin. * Adjusted *p* ≤ 0.1 vs. control food, except for fecal score, for which adjusted *p* ≤ 0.05 vs. control food.

**Table 7:** Digestibility parameters of the study foods.

		**Food with test Ingredient**		
**Parameter**	**Control Food**	**15%**	**30%**	**40%**
True protein digestibility	92.2 ± 6.1	91.1 ± 3.2	93.5 ± 1.6	91.4 ± 1.8
Apparent protein digestibility	88.5 ± 5.7	87.1 ± 2.7	88.0 ± 1.0	86.7 ± 2.1
Apparent fat digestibility	92.4 ± 2.6	85.9 ± 2.4 *	84.0 ± 1.9 *	81.0 ± 2.7 *
Apparent crude fiber digestibility	9.7 ± 8.3	4.2 ± 11.0	4.1 ± 12.8	−5.0 ± 16.0
Apparent neutral detergent fiber digestibility	18.6 ± 6.8	5.0 ± 12.5	18.8 ± 14.9	24.8 ± 12.3
Apparent dry matter digestibility	79.3 ± 2.8	80.7 ± 2.7	83.2 ± 1.1 *	85.0 ± 2.1 *
Food digestible energy, kcal/kg	3610.9 ± 127.4	3661.7 ± 125.1	3693.6 ± 35.7	3705.8 ± 84.8
Food gross energy, kcal/kg	4361.2 ± 0	4383.3 ± 0 *	4339.2 ± 0 *	4295.1 ± 0 *
Food metabolizable energy, kcal/kg	3339.9 ± 110.8	3402.7 ± 117.2	3420.8 ± 35.1	3438.4 ± 78.4

Data are presented as mean ± standard deviation. Values are % unless otherwise indicated. * Adjusted *p* ≤ 0.05 vs. control food.

## Data Availability

The original contributions presented in this study are included in the article/Supplementary Materials. Further inquiries can be directed to the corresponding author.

## Supplementary Materials

The following supporting information can be downloaded at https://www.mdpi.com/article/10.3390/ani15030427/s1, Table S1: Hill’s Pet Nutrition body condition score description; Table S2: Body composition measures by DEXA analyses of the dogs fed four treatment foods over a 182-day period; Table S3: Hematology of healthy adult dogs fed four treatment foods over a 182-day period; Table S4: Serum biochemistry, whole blood taurine, and immune parameters of healthy adult dogs fed four treatment foods over a 182-day period; Table S5: Urinary chemistry of the samples collected from healthy adult dogs fed four treatment foods over a 182-day period; Table S6: Urinary physical characteristics and chemistry analyzed by dipstick method of healthy adult dogs fed four treatment foods over a 182-day period; Table S7: Urinary crystal analysis of healthy adult dogs fed four treatment foods over a 182-day period; Table S8: Urinary sedimental analyses by microscopic examination in healthy adult dogs fed four treatment foods over a 182-day period; Table S9: Urinary casts of healthy adult dogs fed four treatment foods over a 182-day period.
